# Post‐Intensive Care Syndrome: A Growing and Under‐Recognised Condition

**DOI:** 10.1111/jan.16995

**Published:** 2025-04-19

**Authors:** Yuan Chu, David R. Thompson, Josef Trapani, Fiona Timmins

**Affiliations:** ^1^ School of Nursing, Midwifery and Health Systems University College Dublin Dublin Ireland; ^2^ School of Nursing and Midwifery Queen's University Belfast Belfast UK; ^3^ Faculty of Health Sciences University of Malta L‐Imsida Malta

Despite intensive care significantly improving short‐term mortality and 28‐day survival rates (Shi et al. 2024), a growing number of people discharged from intensive care units (ICUs) experience long‐lasting impairments to their physical, cognitive, social and psychological health, which are often under‐recogonised. To raise awareness of these issues among clinicians, patients and their families, the Society of Critical Care Medicine (SCCM) convened a multi‐stakeholder meeting in 2010, coining the term ‘Post‐Intensive Care Syndrome’ (PICS) to conceptualise these long‐term impairments (Needham et al. 2012). However, the features of this syndrome are often diffuse and varied, hampering diagnostic and treatment efforts.

To address this gap, we conducted a concept analysis of PICS (Yuan et al. 2021) and found that the syndrome was characterised by a wide variety of types and degrees of severity of cognitive, psychological, social and physical symptoms following a critical illness that persists beyond hospital discharge and endures up to years after. Common manifestations include generalised weakness, fatigue, reduced mobility, pain, anxiety, depression, sleep disturbances, memory problems, poor concentration, social isolation and so on. The long‐term consequences of PICS are often profoundly disruptive, impacting individual health and well‐being as well as family and social dynamics. Many PICS patients struggle to resume independence, employment and social interaction. The significant loss of independence and increased reliance on caregivers, often family members, places a substantial burden on the patient and their support systems. Thus, PICS is increasingly recognised as a significant public health concern, compounded by the surge in the ageing population and its potential to contribute to the increase in ICU admissions.

To address this concern, establishing robust screening opportunities is paramount, as is integrating the latest technologies and interventions that improve survival, health and well‐being for those with life‐threatening illnesses. As PICS is still a relatively novel and under‐addressed challenge, the absence of standardised outcome measures has hindered clinical and research advancements in this field. The SCCM stakeholders have recommended serial assessments using various tools to evaluate PICS (Mikkelsen et al. 2020). In contrast, another expert consensus has suggested an initial brief screening, followed by a more comprehensive assessment if the initial screening indicates significant PICS impairments (Spies et al. 2021). While both recommendations aim to standardise PICS assessment, they present notable differences in their approaches and the selection of outcome measure instruments. The recommended various tools are also often burdensome for patients, as they are time‐consuming to administer, score, and interpret. Furthermore, the validity and reliability of these measures have been demonstrated in a limited number of studies, raising concerns about their generalisability and applicability in clinical settings. Future efforts should strike a balance between comprehensiveness and practicality, ensuring that the instruments used are both practical and feasible in real‐world clinical practice.

Preventing and mitigating PICS requires a multifaceted approach, beginning with early interventions within the ICU itself. Nurses, as frontline caregivers, are uniquely positioned to provide continuous support and implement strategies to prevent PICS, such as the ABCDEF bundle (**A**ssess, prevent, and manage pain, **B**oth spontaneous awakening trials and spontaneous breathing trials, **C**hoice of analgesia and sedation, **D**elirium assessment, prevention, and management, **E**arly mobility and exercise and **F**amily engagement and empowerment) (Barnes‐Daly et al. 2018), to optimise patient outcomes. Beyond administering optimal analgesia, promoting safe, light sedation practices is essential. Light sedation enables patients to engage more readily with their environment, reducing anxiety and stress while allowing clinicians to maintain closer proximity for reassurance and support (Needham et al. 2012). Additionally, family involvement and visitation are also keystones to effective PICS prevention. Providing information about PICS to family members strengthens the connection between patients, families and medical staff, fostering a supportive environment conducive to recovery. Early and frequent mobilisation is essential for improving physical function in critically ill patients and minimising the impact of ICU treatments. Furthermore, effective care transitions are vital in addressing PICS. Following ICU discharge, patients may transition to various settings, including step‐down care wards, rehabilitation facilities or long‐term acute care hospitals. Seamless care transitions, facilitated by adequate information transfer between providers, are crucial for ensuring a continuum of care and addressing the multidimensional needs of PICS patients. Sharing information about PICS during care transitions ensures that all members of the care team are aware of the patient's needs and contribute to a seamless and coordinated recovery plan.

Recovery from PICS is a continuous journey that extends beyond hospital discharge. Rehabilitation facilities play a vital role in the continuity of care for PICS patients. ICU follow‐up clinics, as an option, offer a specialised setting for evaluating and treating PICS in ICU survivors. Given the complex nature of PICS, leveraging the expertise and resources of rehabilitation institutions and specialists to address the psychological, social, cognitive, and physical impairments associated with PICS is crucial. By providing these essential services, rehabilitation facilities alleviate the burden on acute hospitals and significantly contribute to the care of a substantial number of patients recovering from critical illnesses. The current capacity of in‐ and outpatient rehabilitation services is insufficient, and policymakers need to anticipate the growing need for PICS rehabilitation and allocate additional resources accordingly.

PICS presents tremendous challenges for people transitioning from the ICU. Competently acknowledging the presence of PICS, routine screening for it, and timely prevention and intervention will support a meaningful continuum of care for ICU survivors, enhance their functional reconciliation, and facilitate recovery. Nurses, as primary caregivers, can play a significant role in the continuum of care. By proactively identifying PICS early, implementing preventative measures such as the ABCDEF bundle, educating patients and families to empower their active participation, coordinating holistic care with a multidisciplinary team both within and outside the ICU, and advocating for necessary resources, nurses can optimise the recovery journey for ICU survivors.

## Disclosure

Declaration of AI use: The authors have nothing to report.

## Conflicts of Interest

The authors declare no conflicts of interest.

## Data Availability

The authors have nothing to report.
